# Metabolic engineering of *Corynebacterium glutamicum* for acetate-based itaconic acid production

**DOI:** 10.1186/s13068-022-02238-3

**Published:** 2022-12-14

**Authors:** Marc Schmollack, Felix Werner, Janine Huber, Dirk Kiefer, Manuel Merkel, Rudolf Hausmann, Daniel Siebert, Bastian Blombach

**Affiliations:** 1 Microbial Biotechnology, Campus Straubing for Biotechnology and Sustainability, Technical University of Munich, Uferstraße 53, 94315 Straubing, Germany; 2 Institute of Food Science and Biotechnology, Department of Bioprocess Engineering, University of Hohenheim, Stuttgart, Germany; 3 SynBiofoundry@TUM, Technical University of Munich, Straubing, Germany

**Keywords:** Itaconic acid, *Corynebacterium glutamicum*, Acetic acid, Metabolic engineering, RamB, Glutamate dehydrogenase, Nitrogen limitation, Pyrolysis water

## Abstract

**Background:**

Itaconic acid is a promising platform chemical for a bio-based polymer industry. Today, itaconic acid is biotechnologically produced with *Aspergillus terreus* at industrial scale from sugars. The production of fuels but also of chemicals from food substrates is a dilemma since future processes should rely on carbon sources which do not compete for food or feed. Therefore, the production of chemicals from alternative substrates such as acetate is desirable to develop novel value chains in the bioeconomy.

**Results:**

In this study, *Corynebacterium glutamicum* ATCC 13032 was engineered to efficiently produce itaconic acid from the non-food substrate acetate. Therefore, we rewired the central carbon and nitrogen metabolism by inactivating the transcriptional regulator RamB, reducing the activity of isocitrate dehydrogenase, deletion of the *gdh* gene encoding glutamate dehydrogenase and overexpression of *cis-*aconitate decarboxylase (CAD) from *A. terreus* optimized for expression in *C. glutamicum.* The final strain *C. glutamicum* Δ*ramB* Δ*gdh* IDH^R453C^ (pEKEx2-*malE*cad_opt_) produced 3.43 ± 0.59 g itaconic acid L^−1^ with a product yield of 81 ± 9 mmol mol^−1^ during small-scale cultivations in nitrogen-limited minimal medium containing acetate as sole carbon and energy source. Lowering the cultivation temperature from 30 °C to 25 °C improved CAD activity and further increased the titer and product yield to 5.01 ± 0.67 g L^−1^ and 116 ± 15 mmol mol^−1^, respectively. The latter corresponds to 35% of the theoretical maximum and so far represents the highest product yield for acetate-based itaconic acid production. Further, the optimized strain *C. glutamicum* Δ*ramB* Δ*gdh* IDH^R453C^ (pEKEx2-*malE*cad_opt_), produced 3.38 ± 0.28 g itaconic acid L^−1^ at 25 °C from an acetate-containing aqueous side-stream of fast pyrolysis.

**Conclusion:**

As shown in this study, acetate represents a suitable non-food carbon source for itaconic acid production with *C. glutamicum*. Tailoring the central carbon and nitrogen metabolism enabled the efficient production of itaconic acid from acetate and therefore this study offers useful design principles to genetically engineer *C. glutamicum* for other products from acetate.

**Supplementary Information:**

The online version contains supplementary material available at 10.1186/s13068-022-02238-3.

## Introduction

Biotechnology is considered as a key technology in the bioeconomy to replace the production of chemicals and fuels from fossil raw materials. However, most currently applied biotechnological processes use food substrates such as starch or sugars [1]. This is critically discussed because the ‘fuel versus food’ debate also pertains to the bio-based production of chemicals [2, 3]. Acetate is an alternative carbon source and can be generated from non-food resources such as lignocellulose or C1 gases by pyrolysis, saccharification or anaerobic fermentation using acetogenic bacteria [1, 4]. The valorization of acetate as sole carbon source by microbial fermentation has rarely been addressed so far and research has been primarily focused on the exploitation of *Escherichia coli* as host [1, 4, 5]. The use of acetate as substrate for microbial production processes is strongly limited due to growth inhibition or even toxic properties at elevated concentrations of this weak acid, which can diffuse across the membrane at neutral pH [1].

**Fig. 1:**
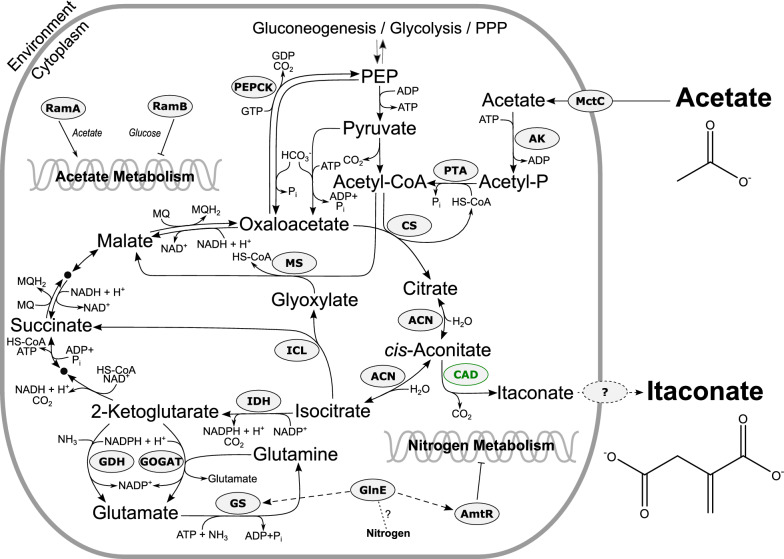
Central metabolism of *C. glutamicum* including pathways relevant for this study. Heterologously expressed CAD is marked in green. Dashed lines indicate unknown or indirect interactions. Abbreviations: *ACN* aconitase; *AK* acetate kinase; *AmtR* global repressor in the nitrogen regulation system; *CAD cis*-aconitate decarboxylase; *CoA* coenzyme A; *CS* citrate synthase; *GDH* glutamate dehydrogenase; *GlnE* adenylyltransferase; *GOGAT* glutamine oxoglutarate aminotransferase; *GS* glutamine synthetase; *ICL* isocitrate lyase; *IDH* isocitrate dehydrogenase; *MctC* monocarboxylic acid transporter; *MQ* menaquinone; *MS* malate synthase; *PEP* phosphoenolpyruvate; *PEPCk* PEP-carboxykinase; *PTA* phosphotransacetylase; *PPP* pentose phosphate pathway; *RamA* regulator of acetate metabolism A; *RamB* regulator of acetate metabolism B

A promising host to valorize acetate is *Corynebacterium glutamicum*, which is an established workhorse for the large-scale production of several amino acids such as l-lysine and l-glutamate in millions of tons per year from sugar-based carbon sources [6]. This Gram-positive bacterium is robust, generally recognized as safe (GRAS), and shows no growth inhibition up to 200 mM of acetate [7]. Several studies already utilized *C. glutamicum* for the production of chemicals such as L-valine [8, 9], L-lysine [10], ketoisovalerate [11], isobutanol [12] and 1,2-propanediol [13] with acetate as co-substrate for biomass formation. Furthermore, Kiefer et al*.* [14] established an acetate-based fed-batch process for high cell density cultivation of *C. glutamicum* ATCC 13032 on acetate as single carbon and energy source*,* yielding biomass concentrations of up to 80 g L^−1^ with a maximal growth rate (µ) of 0.39 h^−1^. More recently, the same authors used this process to demonstrate high-level production of recombinant proteins (2.7 g L^−1^) by *C. glutamicum* [15] paving the way for large-scale processes with this bacterium on acetate as sole carbon and energy source. The acetate metabolism of *C. glutamicum* and its regulation have been extensively studied [16–18]. As described above, acetic acid enters the cell by diffusion, but is also actively transported by the monocarboxylic acid transporter (MctC) [19]. Inside the cell, acetate is activated by the action of acetate kinase (AK) and phosphotransacetylase (PTA) to acetyl-CoA which is further metabolized via the TCA cycle to isocitrate [16]. During growth on acetate, about one-third of isocitrate is channeled into the essentially required glyoxylate shunt catalyzed by the enzymes isocitrate lyase (ICL) and malate synthase (MS). The residual isocitrate is oxidized by the enzymes of the TCA cycle [20]. The eventually formed oxaloacetate is partially converted to phosphoenolpyruvate (PEP) by PEP carboxykinase (PEPCk) for the formation of pyruvate and gluconeogenesis [16]. The transcription of genes related to the acetate metabolism is mainly controlled by the transcriptional regulators RamA and RamB. RamA was identified as an essential transcriptional activator during growth on acetate or ethanol, whereas RamB was found to function as a repressor in the presence of glucose [18, 21] (Fig. 1).

Itaconic acid (IA) is an unsaturated dicarboxylic acid and was suggested in 2004 as one of 12 promising candidates for biotechnologically produced crude chemicals [22]. In 2010, IA was removed from this list due to remittent attention in the scientific literature [23]. Since then, IA has regained attraction, as evidenced by the steadily increasing number of publications and patents [24, 25]. Among other applications, IA is mainly used as a co-polymer in hydrogels and resins for drug delivery systems and water treatment [24]. As an immunomodulatory compound with antimicrobial activity, IA has gained further interest in clinical research [26] as well. Due to the unique structural properties of IA, it is predicted to be an environmentally friendly and sustainable substitute for (meth-) acrylic acid in plastic production [27, 28].

At the industrial level, IA is biotechnologically produced from sugar or sugar-associated substrates such as starch and molasses using the filamentous fungus *Aspergillus terreus* [29]. The currently applied processes yield high titers (about 160 g L^−1^) but they require careful process control due to the sensitivity of the fungus to e.g., shear stress, and show considerable low and therefore improvable productivities [30]. Consequently, alternative microbial hosts such as *Ustilago maydis*, *E. coli* or *C. glutamicum* have been engineered for IA production but mainly from glucose as carbon source [31–33]. Recently, we engineered *C. glutamicum* for IA production from acetate as sole carbon source by expression of the *cis*-aconitate decarboxylase from *A. terreus* and attenuation of isocitrate dehydrogenase (IDH) activity. In fed-batch fermentations with an adjusted C:N ratio, the engineered *C. glutamicum* IDH^R453C^ (pEKEx2-*malE*cad_opt_) strain produced 29 g IA L^−1^ with a peak volumetric productivity (Q_P_) of 1 g L^−1^ h^−1^ and a product yield (Y_P/S_) of 0.16 g IA per g of acetate [34]. To further improve the production of IA from acetate with *C. glutamicum*, we systematically engineered the acetate metabolism and its regulation, perturbed the nitrogen homeostasis and optimized the culture conditions. Finally, we validated the performance of the newly constructed strain in the biorefinery side-stream pyrolysis water, which contains significant amounts of acetate.

## Results

### Enabling initial itaconic acid production from acetate

*C. glutamicum* is naturally unable to produce IA. However, heterologous plasmid-based expression of the codon optimized gene of the *cis-*aconitate decarboxylase (CAD) from *A. terreus*, stabilized by fusion to the maltose binding protein (MalE) derived from *E. coli* was already successfully applied for IA production with *C. glutamicum* from glucose [33]. The plasmid pEKEx2-*malEcad*_opt_ was transformed into *C. glutamicum* ATCC 13032 and the resulting strain *C. glutamicum* ITA1 was cultured in small-scale (1 mL) cultures in CGXII medium supplemented with 20 g acetate L^−1^. However, no IA could be detected in the culture medium after 72 h. Otten et al*.* [33] showed that application of nitrogen limitation increased IA production of *C. glutamicum* from glucose, hence, we reduced the nitrogen concentration in the medium from a C:N ratio of 2.75:1 to 40:1 [33, 34]. By applying nitrogen-limiting conditions, *C. glutamicum* ITA1 produced 0.32 ± 0.09 g IA L^−1^ after 72 h, corresponding to a Y_P/S_ of 8 ± 2 mmol IA per mol acetate (Table 1). Due to the rather low affinity of CAD towards its substrate *cis*-aconitate (K_M_ of 2.45 mM) [35], reducing carbon flux via IDH is a common strategy for IA production in bacterial hosts [33, 36, 37]. Therefore, we exchanged the native *icd* gene in *C. glutamicum* ITA1 by three versions that code for IDH^A94D^, IDH^G407S^ and IDH^R453C^, which possess 10, 55 and 29% of the wild type IDH activity, respectively [38]. *C. glutamicum* IDH^A94D^ (pEKEx2-*malEcad*_opt_), *C. glutamicum* IDH^G407S^ (pEKEx2-*malEcad*_opt_) and *C. glutamicum* IDH^R453C^ (pEKEx2-*malEcad*_opt_), designated as *C. glutamicum* ITA2-4, were cultivated as described above and the IA titer and the Y_P/S_ were determined after 72 h. *C. glutamicum* ITA2 and ITA3 reached titers of 0.35 ± 0.06 g L^−1^ and 0.49 ± 0.18 g L^−1^ with a Y_P/S_ of 9 ± 2 mmol mol^−1^ and 13 ± 6 mmol mol^−1^, respectively. *C. glutamicum* ITA4 showed the highest Y_P/S_ and titer of 20 ± 5 mmol mol^−1^ and 0.75 ± 0.18 g L^−1^ (Table 1).

**Table 1:** Yields (Y_P/S_) and titers of engineered *C. glutamicum* strains cultivated as 1 mL cultures

Strain	Genotype	Y_P/S_, mmol mol^−1^	Titer, g L^−1^
ITA1	(pEKEx2-*malEcad*_opt_)	8 ± 2	0.32 ± 0.09
ITA2	IDH^A94D^ (pEKEx2-*malEcad*_opt_)	9 ± 2	0.35 ± 0.06
ITA3	IDH^G407S^ (pEKEx2-*malEcad*_opt_)	13 ± 6	0.49 ± 0.18
ITA4	IDH^R453C^ (pEKEx2-*malEcad*_opt_)	20 ± 5	0.75 ± 0.18
ITA5	IDH^R453C^ P_*pck*_::P_*dapA*_-A8 (pEKEx2-*malEcad*_opt_)	10 ± 1	0.39 ± 0.00
ITA6	IDH^R453C^ P_*pck*_::P_*dapA*_-A16 (pEKEx2-*malEcad*_opt_)	19 ± 4	0.73 ± 0.13
ITA7	IDH^R453C^ P_*pck*_::P_*dapA*_-C5 (pEKEx2-*malEcad*_opt_)	No growth	
ITA8	IDH^R453C^ P_*pck*_::P_*dapA*_-C7 (pEKEx2-*malEcad*_opt_)	12 ± 1	0.39 ± 0.00
ITA9	Δ*ramB* (pEKEx2-*malEcad*_opt_)	36 ± 4	1.54 ± 0.18
ITA10	Δ*ramB* (pEKEx2-*malEcad*_opt_-*ramA*)	39 ± 6	1.35 ± 0.23
ITA11	Δ*ramB* Δ*glnE* (pEKEx2-*malEcad*_opt_)	18 ± 4	0.60 ± 0.21
ITA12	Δ*ramB* Δ*gdh* (pEKEx2-*malEcad*_opt_)	62 ± 12	2.36 ± 0.75
ITA13	Δ*ramB* Δ*gdh* IDH^R453C^ (pEKEx2-*malEcad*_opt_)	81 ± 9	3.43 ± 0.59

### Engineering key metabolic pathways of acetate metabolism

During growth of *C. glutamicum* on acetate, gluconeogenesis and the glyoxylate shunt are highly active compared to growth on glycolytic substrates such as glucose [20]. Both pathways are essential for growth on acetate [39, 40], but also form additional carbon sinks (Fig. 1). We speculated that a reduction of PEPCk activity might lead to a tailback of metabolites in the TCA thereby increasing the precursor availability for IA production. Therefore, the native promotor of the *pck* gene coding for PEPCk was exchanged in *C. glutamicum* ITA4 with different variants of a *dapA-*promoter library [41]. However, the generated strains *C. glutamicum* ITA5-8 that carry the promoter versions P_*dapA*_-A8, -A16, -C5 and -C7, respectively, showed similar or even reduced product yields and IA titers compared to the parental strain *C. glutamicum* ITA4 (Table 1). No growth at all could be observed for *C. glutamicum* ITA7 carrying the P_*dpaA*_*-*C5 version which should yield the lowest promoter activity among the tested variants [41]. We also tested a complete inactivation of PEPCk on IA production and deleted the *pck* gene in *C. glutamicum* ITA1. As expected, the resulting strain was unable to grow with acetate as sole carbon source, and the resting cells were metabolically inactive and neither consumed any acetate nor produced any IA.

The first step of the glyoxylate shunt is catalyzed by ICL, which converts isocitrate to succinate and glyoxylate [40]. Although during growth on acetate the carbon flux over ICL is three times lower compared to the flux via IDH [20], the glyoxylate shunt forms a considerable sink for isocitrate. Similar to reduced IDH activity, a reduced activity of ICL might cause a backlog of isocitrate, which in turn would increase the pool of *cis*-aconitate and positively affect IA production. It was shown that during growth on acetate, RamB is required to fully activate transcription of the *aceA* and *aceB* genes encoding ICL and MS and deletion of the *ramB* gene strongly reduced ICL activity in *C. glutamicum* [42]. Therefore, to reduce carbon flux through the glyoxylate-shunt, the *ramB* gene was deleted in *C. glutamicum* ITA1, thus generating *C. glutamicum* Δ*ramB* (pEKEx2-*malEcad*_opt_) named *C. glutamicum* ITA9. Although the growth rate of *C. glutamicum* ITA9 (0.12 ± 0.01 h^−1^) was reduced by 20% compared to *C. glutamicum* ITA1 and ITA4, the Y_P/S_ and the IA titer increased to 36 ± 4 mmol mol^−1^ and 1.54 ± 0.18 g L^−1^, in 1 mL cultures after 72 h (Table 1). It is noteworthy that without reducing IDH activity, *C. glutamicum* ITA9 showed an 80% higher Y_P/S_ compared to *C. glutamicum* ITA4. In addition to the deletion of *ramB*, *ramA* was overexpressed in *C. glutamicum* ITA9 as it was shown that acceleration of the acetate metabolism was beneficial for IA production from acetate with a tailored *E. coli* strain [43]. However, compared to the parental strain *C. glutamicum* ITA9, the Y_P/S_ of *C. glutamicum* Δ*ramB* (pEKEx2-*malEcad*_opt_-*ramA*) (*C. glutamicum* ITA10) was not significantly increased and the final titer was even slightly reduced to 1.35 ± 0.23 g L^−1^ (Table 1).

### Engineering nitrogen homeostasis

As described above, the application of nitrogen-limiting conditions is essential for the production of IA from acetate with *C. glutamicum*. To mimic intracellular nitrogen limitation, we inactivated the adenylyltransferase GlnE in *C. glutamicum* ITA9, generating *C. glutamicum* Δ*ramB* Δ*glnE* (pEKEx2-*malEcad*_opt_) (*C. glutamicum* ITA11). GlnE is involved in controlling nitrogen homeostasis, and the deletion of the corresponding *glnE* gene in the wild type causes a nitrogen limitation response at the transcriptional level [44] (Fig. 1). However, compared to *C. glutamicum* ITA9, the Y_P/S_ and the IA titer of the *glnE* knock-out strain were strongly diminished (Table 1). As an alternative approach, we directly inactivated glutamate dehydrogenase, which forms the major nitrogen assimilation pathway in *C. glutamicum* under nitrogen excess (Fig. 1), by deletion of the *gdh* gene in *C. glutamicum* ITA9. The newly constructed strain *C. glutamicum* Δ*ramB* Δ*gdh* (pEKEx2-*malEcad*_opt_) (*C. glutamicum* ITA12) showed a Y_P/S_ and IA titer of 62 ± 12 mmol mol^−1^ and 2.36 ± 0.75 g L^−1^ if cultivated under nitrogen limitation. This corresponds to an increase of 72% compared to *C. glutamicum* ITA9 (Table 1). It is noteworthy that neither *C. glutamicum* ITA11 nor ITA12 secreted any IA in small-scale cultivations under nitrogen excess, and even a combination of the *gdh* and *glnE* deletion did not lead to IA production under a nitrogen surplus.

### Combining beneficial mutations

To combine the beneficial mutations, the native *icd* of strain ITA12 was exchanged with the mutated version coding for IDH^R453C^ of *C. glutamicum* ITA4. Y_P/S_ and IA titer of *C. glutamicum* Δ*ramB* Δ*gdh* IDH^R453C^ (pEKEx2-*malEcad*_opt_) (*C. glutamicum* ITA13) further increased to 81 ± 9 mmol mol^−1^ and 3.43 ± 0.59 g L^−1^, respectively (Table 1). The applied metabolic engineering approach increased the Y_P/S_ and IA titer of *C. glutamicum* ITA13 compared to *C. glutamicum* ITA1 tenfold, to 24% of the theoretical yield of 333 mmol IA per mole acetate [43].

### Reduction of the cultivation temperature improves IA production

**Fig. 2:**
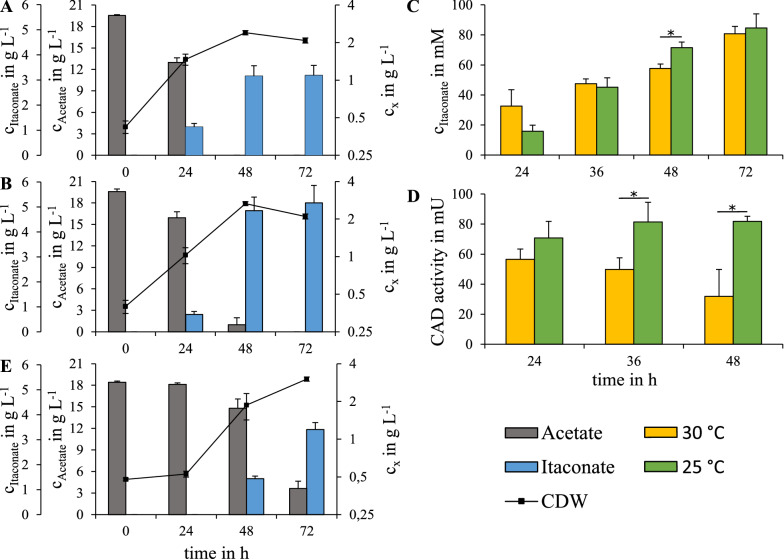
Shaking flask cultivations of *C. glutamicum* ITA13. **A** cultivation at 30 °C; **B** cultivation at 25 °C. **C** intracellular concentrations of IA and **D** CAD activity during cultivation at 30 °C and 25 °C. **E** cultivation with 50% (v/v) PW as substrate at 25 °C. The asterisk indicates significance according to a two-sample t-test (*p* < 0.05)

Vuoristo et al*.* [45] showed that lowered cultivation temperatures increased CAD activities and IA titers in *E. coli*. Consequently, we compared the performance of *C. glutamicum* ITA13 in 50 mL shaking-flask cultures at 30 °C and 25 °C for 72 h. Under both conditions, *C. glutamicum* ITA13 reached a maximal biomass concentration of about 2.5 g L^−1^ after 48 h. At 30 °C, the acetate was completely consumed after 48 h, whereas at 25 °C, 1.59 ± 0.63 g acetate L^−1^ were still present but were subsequently metabolized (Fig. 2A, B). After 72 h of cultivation, *C. glutamicum* ITA13 produced 3.19 ± 0.40 g IA L^−1^ with a Y_P/S_ of 74 ± 10 mmol mol^−1^ at 30 °C. At 25 °C, this strain showed a significantly increased titer of 5.01 ± 0.67 g IA L^−1^ with a Y_P/S_ of 116 ± 15 mmol mol^−1^; a 57% increase compared to the cultivation at 30 °C, which corresponds to 35% of the theoretical maximum.

In order to understand the positive effect on IA production by a decrease of the cultivation temperature, we determined the intracellular concentrations of IA and the CAD activity of *C. glutamicum* ITA13 at 30 °C and 25 °C. Intracellular concentrations of IA exceeded extracellular concentrations at all measured time points independently of the cultivation temperature and reached levels of > 80 mM after 72 h (Fig. 2C). The CAD activity of *C. glutamicum* ITA13 at 30 °C was highest after 24 h with 57 ± 7 mU and steadily decreased to 32 ± 18 mU after 48 h. In contrast, at 25 °C the strain possessed 25% increased activity of 71 ± 11 mU, which further increased to 82 ± 3 mU after 48 h (Fig. 2 D).

### Production of itaconic acid from aqueous side-stream of fast pyrolysis

The aqueous side-stream (named pyrolysis water, PW) generated by fast pyrolysis of straw, in the bioliq® process, has a water content of around 80% (w/w) and besides several other compounds, it contains about 40 g acetate L^−1^ [46]. However, crude PW was found to inhibit growth of *C. glutamicum* ITA13 at concentrations below 2% (v/v) in CGXII medium. In order to increase biocompatibility, the crude PW was treated by over-liming, vacuum evaporation, heat- and activated carbon treatment as described by Kubisch and Ochsenreither [47]. The processed PW was used as substrate for cultivation of *C. glutamicum* ITA13 in nitrogen-limited CGXII medium in 50 mL shaking-flask cultures at 25 °C. The PW was mixed with modified CGXII medium to a final concentration of 50% (v/v) in order to reach a starting concentration of 18 ± 0.17 g acetate L^−1^. Under these conditions, *C. glutamicum* ITA13 showed a lag-phase of 24 h, but reached a final biomass concentration of 3.32 ± 0.11 g L^−1^ after 72 h. In contrast to cultures supplemented with pure acetate, 3.64 ± 1.02 g acetate L^−1^ remained in the culture supernatant after 72 h. IA production started after 24 h and *C. glutamicum* ITA13 produced 3.38 ± 0.28 g IA L^−1^ after 72 h, which corresponds to a yield of 104 ± 4 mmol itaconic acid per mole of consumed acetate (Fig. 2E). However, the actual yield is below the calculated yield since PW contains, besides acetate, other potential substrates that can be used by *C. glutamicum*. HPLC analysis revealed 1.83 ± 0.00 g propionate L^−1^ which were completely consumed by *C. glutamicum* ITA13 during 72 h of cultivation. Total carbon (TC) measurements showed that the concentration of total organic carbon (TOC) amounted to 18.25 ± 0.17 g carbon L^−1^ at the beginning of the cultivation. A fraction of 8.22 ± 0.09 g L^−1^ originated from the MOPS buffer in the CGXII medium. The remaining 10.03 ± 0.18 g TOC L^−1^ originate from the PW and consisted of 7.49 ± 0.07 g carbon_Acetate_ L^−1^, 0.90 ± 0.00 g carbon_Propionate_ L^−1^ and 1.63 ± 0.11 g carbon_Unknown_ L^−1^ (the concentrations of acetate and propionate were determined by HPLC). Within 72 h, *C. glutamicum* ITA13 converted 15% of the TOC in PW to IA, whereas 29% of g carbon_Acetate_ L^−1^ (calculated from HPLC data) was converted to IA in cultures supplemented with acetate as sole carbon source.

## Discussion

Acetate is a promising alternative non-food derived feedstock for industrial biotechnology. Since *C. glutamicum* shows almost the same µ and carbon uptake rates during growth on acetate compared to growth on glucose [20] and tolerates high concentrations of IA without degrading this organic acid [33], we systematically engineered this bacterium for the production of IA from acetate as sole carbon and energy source. In contrast to the study performed by Otten et al*.* with glucose as substrate [33], the exclusive overexpression of the optimized *cad* construct in *C. glutamicum* ITA1 did not enable IA production from acetate. Only after applying nitrogen-limiting conditions, *C. glutamicum* ITA1 started to secrete this dicarboxylic acid. Since the K_M_ value of 18.5 µM for *cis*-aconitate of aconitase [48] is more than 100-fold lower compared to the K_M_ value of the CAD enzyme (2.45 mM) from *A. terreus* [35], we reduced IDH activity to increase the availability of *cis*-aconitate for the CAD enzyme. The highest IA yield was observed for the IDH version R453C with 29% of wild type activity, while the stronger and weaker versions A94D and G407S with 10 and 55% residual activity [38] only slightly improved IA production from acetate compared to ITA1. In contrast, a residual IDH activity of only 3% proved to be most suitable for IA production from glucose [33]. This discrepancy might be explained by the altered carbon flux distribution during growth on acetate compared to glucose [20]. Due to the threefold lower carbon flux over IDH during growth on glucose, a stronger reduction of IDH activity might be required to sufficiently improve *cis*-aconitate availability for IA production. During growth on acetate, the glyoxylate shunt is essential and forms a second sink of isocitrate [40]. To further improve *cis*-aconitate availability, we deleted the *ramB* gene encoding the transcriptional regulator RamB to reduce the expression of ICL and MS [42] and therefore the carbon flux via the glyoxylate shunt. Interestingly, IA titers reached by *C. glutamicum* ITA9 with inactivated RamB doubled compared to the *C. glutamicum* ITA4 with reduced activity of IDH, although the carbon flux over IDH is three times higher compared to the flux over ICL in the wild type of *C. glutamicum* during growth on acetate [20]. This unexpected improvement of the Y_P/S_ by the deletion of *ramB* indicates that, as a result of the reduced IDH activity, the carbon flux via the glyoxylate shunt increased in *C. glutamicum* ITA4. However, it should be stated that RamB is a global regulator of the carbon metabolism in *C. glutamicum* [17, 18, 21] and the role of RamB during growth on acetate as sole carbon source has not been fully investigated in detail. Therefore, additional regulatory effects other than the reduction of the carbon flux via the glyoxylate shunt might positively affect IA production in *C. glutamicum* ITA9. Moreover, it should be considered that IA is a potent inhibitor of ICL in different species and also inhibits the purified ICL enzyme of *C. glutamicum* in a linear uncompetitive manner with a K_*i*_ of 5.05 µM [40]. Indeed, non-induced cultures showed higher growth rates compared to induced cultures in our experimental set-up. Furthermore, we observed that µ of *C. glutamicum* wild type in minimal medium on acetate was reduced to 0.26 ± 0.01 h^−1^ by the addition of 50 mM IA, and completely abolished by the addition of 250 mM IA compared to 0.31 ± 0.01 h^−1^ without the addition of IA at 30 °C (Additional file 1: Figure S1). Interestingly, the effect of ICL inhibition by IA seems to be less pronounced in our production strains compared to in vitro studies, as the engineered strains intracellularly accumulated up to 80 mM IA during IA production. Vice versa, the high intracellular IA concentrations might contribute to ICL inhibition and, as described above, consequently to improved IA production. However, ICL variants insensitive to IA might be useful to further improve IA production from acetate with *C. glutamicum*.

Appling nitrogen limitation during IA production from acetate was essential in our experimental set-up. To mimic an intracellular nitrogen limitation, we inactivated glutamate dehydrogenase (GDH) which is part of the major nitrogen assimilation pathway in *C. glutamicum* under nitrogen surplus [49]. In fact, the Y_P/S_ of the *gdh* deficient mutant *C. glutamicum* ITA12 increased by 72% compared to *C. glutamicum* ITA9. Deletion of *gdh* in the wild type of *C. glutamicum* was shown to increase the intracellular pool of 2-oxoglutarate [50], which is in the range of the K_i_ for IDH [51], and might also contribute to IDH inhibition. In contrast to *C. glutamicum* ITA12, the Y_P/S_ of the *glnE* knock-out strain *C. glutamicum* ITA11 was reduced by 50% compared to the parental strain *C. glutamicum* ITA9. Rehm et al*.* [44] showed that deletion of *glnE* causes a nitrogen limitation response on the transcriptional level; however, intracellular pools of 2-oxoglutarate were reduced by almost 50%. Our results indicate that increased intracellular pools of 2-oxoglutarate are beneficial for IA production from acetate with *C. glutamicum*. Furthermore, we showed that the identified beneficial mutations could be combined to improve IA production and the tailored strain *C. glutamicum* ITA13 showed a Y_P/S_ of 81 ± 9 mmol mol^−1^. By lowering the cultivation temperature to 25 °C, the Y_P/S_ was further increased to 116 ± 15 mmol mol^−1^, which corresponds to 35% of the theoretical maximum and is the highest yield of itaconic acid produced from acetate as sole carbon and energy source reported so far. The increased Y_P/S_ observed during cultivation at 25 °C is probably not caused by an improved export of IA due to an altered membrane composition compared to 30 °C [52].

Although, *C. glutamicum* ITA13 showed significantly higher intracellular IA concentrations at 25 °C after 48 h, which is probably due to the higher titers produced compared to 30 °C; about 80 mM IA accumulated in the cell after 72 h, independent of the cultivation temperature. These data match the concentrations of intracellular IA found during production of IA from glucose reported by Otten et al*.* [33]. However, cells cultivated at 25 °C showed significantly increased CAD activities compared to cells cultivated at 30 °C. Similar observations were reported by Vuoristo et al*.* [45], who found 12-fold higher CAD activity in IA producing *E. coli* cultured at 30 °C compared to cells cultured at 37 °C. These results suggest that higher CAD activities boost the production of IA. This assumption is further supported by our observations that the genomic integration of the P_*tac*_-*malEcad*_opt_ construct strongly reduced IA production compared to the plasmid carrying derivative strain. Nevertheless, global analysis of transcriptome, metabolome and carbon flux need to be performed in order to fully understand the positive effect of lowering the cultivation temperature during IA production with *C. glutamicum* from acetate.

In addition, we showed that our final strain is able to produce IA from the acetate-containing side-stream of fast pyrolysis. An Y_P/S_ of 104 ± 4 mmol mol^−1^ was reached by *C. glutamicum* ITA12 from 50% processed PW in 72 h. This yield is almost as high as the yield reached on pure acetate. However, only 15% of the TOC in PW were converted to IA, whereas 29% TOC of pure acetate were converted to IA. This discrepancy occurs due to a big fraction of unknown carbon compounds in PW that *C. glutamicum* cannot use as substrate and the remaining acetate after 72 h. Further, some of these unknown compounds are most likely toxic to *C. glutamicum* and therefore responsible for elongated lag-phases compared to cultures containing pure acetate.

## Conclusion

In this study, we tailored *C. glutamicum* for the efficient production of IA from the non-food carbon source acetate by rewiring the central carbon and nitrogen metabolism and optimizing the culture conditions. The product yield of *C. glutamicum* Δ*ramB* Δ*gdh* IDH^R453C^ (pEKEx2-*malE*cad_opt_) (ITA13) is the highest for IA production from acetate as sole substrate reported so far. Furthermore, we proved that this strain is a useful basis to valorize the acetate-containing side-stream of fast pyrolysis. To fully exploit the potential of the engineered strains, future studies will focus on the scale-up of IA production from acetate and pyrolysis water.

## Material and methods

### Bacterial strains, plasmids, and oligonucleotides

All the bacterial strains, plasmids, and oligonucleotides used and engineered in this study as well as their relevant characteristics, sequences and sources or purposes are listed in ‘Additional file 2’.

### Cloning procedures

All enzymes were purchased from New England BioLabs GmbH (Frankfurt am Main, Germany) and used according to the manufacturer’s specifications. Commercial kits for DNA purification—NucleoSpin® Plasmid; NucleoSpin® Gel and PCR Clean-up; NucleoSpin® Microbial DNA—were purchased from Machery-Nagel GmbH & Co. KG (Düren, Germany) and used according to the manufacturer’s protocol. Oligonucleotides were ordered from Sigma-Aldrich Chemie GmbH (Taufkirchen, Germany). Sanger sequencing of plasmids and PCR products was conducted by Microsynth Seqlab GmbH (Göttingen, Germany).

*E. coli* DH5α was used for plasmid amplification and maintenance. The plasmids were purified from a respective overnight culture with the NucleoSpin® plasmid kit and linearized with respective enzymes if necessary. DNA fragments and genes were amplified with Q5® high-fidelity DNA polymerase as recommended by the manufacturer. PCR was controlled by agarose-gel electrophoresis and PCR products and linearized plasmids were purified with the NucleoSpin® Gel and PCR Clean-up kit. The respective DNA fragments were joined via Gibson Assembly [53]. The obtained product was used for transformation of chemical competent *E. coli* DH5α [54]. Eventually, the plasmid sequence was verified by sequencing. The correct plasmids were used to transform electrocompetent *C. glutamicum* [55, 56]. Genomic modifications of *C. glutamicum* were conducted by double homologue cross-over events using the pK19*mobsacB* vector system with kanamycin resistance [57]. The modified strains were verified by colony PCR or sequencing if necessary. All strains were preserved in 30% (v/v) sterile glycerol at − 80 °C.

### Media and cultivation conditions

All chemicals were purchased from Carl Roth GmbH & Co. KG (Karlsruhe, Germany). Bacto™ tryptone and BBL™ yeast extract were purchased from Becton Dickinson GmbH (Heidelberg, Germany).

*E. coli* was cultured in 2YT medium [54] at 37 °C. 18 g agar L^−1^ was added to the medium for preparation of plates. For growth experiments, *C. glutamicum* strains were cultured on solid 2YT plates for two to three days at 30 °C. A single colony was used to inoculate 5 mL of 2YT medium and the culture was incubated for six to eight hours. The whole culture was transferred into 50 mL 2YT medium in 500 mL shaking flasks containing four baffles, closed with a cellulose plug and cultivated for 16 h. The latter culture was used for inoculation of main cultures as either 1 mL small-scale cultivation using a BioLector® system (m2p-labs GmbH; Baesweiler, Germany) or as 50 mL shaking flask cultivations. Therefore, an appropriate volume of the pre-culture was harvested by centrifugation at 4000 g for 10 min at room temperature. The supernatant was discarded and the pellet was resuspended in 0.9% (w/v) NaCl (4% (v/v) of the final volume) prior to inoculation. Main cultures of *C. glutamicum* were cultivated in modified CGXII medium [58] consisting of 5 g urea L^−1^ and 5 g (NH_4_)_2_SO_4_ L^−1^, 1 g K_2_HPO_4_ L^−1^ and 1 g KH_2_PO_4_ L^−1^ and 21 g MOPS L^−1^. Prior to inoculation, the minimal medium was supplemented with MgSO_4_, CaCl_2_ × 2 H_2_O and biotin to reach a final concentration of 250 mg L^−1^, 10 mg L^−1^ and 0.2 mg L^−1^, respectively. All three solutions were prepared as 1000 × concentrated aqueous stock and sterile filtered with a 0.2 µm cellulose acetate syringe filter (Sarstedt AG & Co. KG; 51588 Nürnbrecht, Germany). Additionally, a respective amount of sterile filtered 1000 × concentrated aqueous trace element solution (16.4 g FeSO_4_ × 7 H_2_O; 0.1 g MnSO_4_ x H_2_O; 0.313 g CuSO_4_ × 5 H_2_O; 1 g ZnSO_4_ × 7 H_2_O; 0.02 g NiCl_2_ × 6 H_2_O per liter—the pH adjusted with 32% (w/v) HCl to 1) was added. To apply nitrogen limitations, the C:N ratio was increased to 40:1 by reducing the amount of urea (0.5 g urea L^−1^ for 20 g acetate L^−1^) and omitting (NH_4_)_2_SO_4_ during media preparation. The pH was adjusted to 6.5 with 5 M KOH before autoclaving. Acetate as carbon source was added from an 81.7% (w/v), sterile filtered aqueous potassium acetate solution. The experiments with pyrolysis water (PW) contained 25 mL of processed PW (see below), which was added to the medium. Gene expression from pEKEx2 plasmids was induced by 1 mM IPTG (final concentration) added from a sterile filtered 1000 × aqueous stock. Whenever necessary, plates and liquid cultures were supplemented with 50 µg kanamycin sulfate L^−1^.

BioLector® measurements of the small-scale cultures were conducted in a 48-well flower plate as described before by Siebert et al*.* [59]. 2 mL cultures were inoculated to an OD of 1. 1 mL was harvested prior to incubation by centrifugation at 21,300 g for 10 min at room temperature. The supernatant was subsequently transferred into a new 1.5 mL reaction tube and stored at − 20 °C until further analysis. The remaining culture was incubated at 1000 rpm, 30 °C and 85% humidity for 72 h. After 72 h, the temperature was shifted and held at 10 °C until the cultures were harvested, as described above. The shaking flask cultivations were performed as 50 mL CGXII cultures in 500 mL baffled shaking flasks and closed with cellulose plugs. These cultures were inoculated to an OD_600_ of 2 and grown in a Multitron2® orbital shaker (Infors GmbH; Einsbach, Germany) with an amplitude of 25 mm at 30 °C or 25 °C at 180 rpm. At the respective time points, 1 mL of culture was taken and OD_600_ was measured to determine cell dry weight with a photometer specific correlation factor of 0.23–0.27. The remaining volume was centrifuged and stored for further analysis as described above.

### Measurement of intracellular itaconic acid

For the intracellular measurements of IA, the cells were rapidly separated and lysed by silicon oil centrifugation [60, 61]. 200 µl of culture were transferred to a 1.5 mL reaction tube containing 300 µl silicon oil PN200 floating on a layer of 100 µl 35% (w/v) perchloric acid. The samples were immediately centrifuged at 21300 g for 10 min at RT. After centrifugation, the remaining culture supernatant was carefully removed together with the oil phase. The cell pellet was resuspended in the remaining acidic fraction and neutralized by adding 100 µl of 5.8 M KOH containing 1 M of KH_2_PO_4_. The samples were shortly vortexed and stored on ice for 30 min. After final centrifugation at 15000 g and 4 °C for 30 min, the sample supernatant was filtrated with a 0.45 µm cellulose acetate filter (Agilent Technologies; Waldbronn, Germany) and stored at − 20 °C for further analysis. For calculating intracellular concentrations, a cytoplasmic volume of 1.7 µl mg^−1^ cell dry weight was presumed [33].

### HPLC measurements

Determination of organic acids in the culture supernatants was performed via HPLC as described by Siebert et al*.* [59]. A 1260 Infinity II system (Agilent Technologies; Waldbronn, Germany) was equipped with a Hi-Plex H column (7.7 × 300 mm, 8 µm) together with a Hi-Plex H guard cartridge (3 × 5 mm, 8 µm) heated to 50 °C or 30 °C if the samples contained PW. 5 mM aqueous sulfuric acid was used as mobile phase with a flow rate of 0.4 mL min^−1^ over 35 min or 60 min if the samples contained PW. Signals were acquired by a refractive index detector (RID). The concentrations of intracellular itaconate, as well as itaconic acid produced in the CAD assay (see below) were determined as described above and the signal was acquired via a diode array detector (DAD) at 215 nm.

The received relative units obtained for the RID measurements were calculated to a concentration in mM by measurements of standardized concentrations between 1 and 250 mM of the respective compound. The received relative units obtained for the DAD measurements were calculated to a concentration in µM by measurements of standardized concentrations between 10 µM and 5 mM of the respective compound.

### Total carbon measurement

Measurements of the carbon fractions in the culture supernatants were conducted with a multi N/C 2100S TOC/TN_b_ analyzer (Analytik Jena GmbH; Jena, Germany) according to Buchholz et al*.* [62]. 100 µl of properly diluted culture supernatant were used for the determination of total carbon (TC) by combustion at 800 °C and inorganic carbon (IC) by acidification with 10% (w/v) *o*-phosphoric acid by 3 to 4 technical replicates. The CO_2_ formed during TC and IC measurements was detected by a nondispersive infrared sensor. The relative signal was converted to concentrations in g L^−1^ by a calibration curve within the range of 100 mg L^−1^ to 1.5 g L^−1^ of IC and 400 mg L^−1^ to 3 g L^−1^ TC. The total organic carbon (TOC) was calculated as the difference of TC and IC.

### Determination of CAD activity

For determining CAD activity, 50 mL culture were harvested by centrifugation at 4000 g for 20 min at 4 °C. The cells were washed twice with 25 mL of ice-cold 0.2 M potassium phosphate buffer (pH 6.5). The cells were resuspended in 1 mL buffer and transferred into a 1.5 mL screw-cap tube containing 250 µl of glass beads placed on ice. For cell lysis, a Precellys24® (Bertin Instruments; Montigny-le-Bretonneux, France) cell disrupter was used in cycles of 6 × 30 s interrupted by 2 min on ice. The glass beads were sedimented by short spin in a tabletop centrifuge. The supernatant was transferred to a new 1.5 mL reaction tube and centrifuged at 15,000 g at 4 °C for 30 min to remove cell debris. The enzyme assay was performed as previously described by Otten et al*.* [33]. 100 µl of cell crude extract were added to 900 µl of 0.2 M potassium phosphate buffer containing 8 mM cis-aconitic acid pH 6.2. The samples were shortly vortexed and incubated at 37 °C and 300 rpm in a thermo-shaker. After 10 min, 38 µl of 32% (w/v) HCl was added to the samples to stop the reaction. The samples were vortexed and centrifuged at 21,300 g for 10 min at RT. The supernatant was transferred to a new 1.5 mL reaction tube and stored at − 20 °C until further use.

Enzyme concentrations of crude extracts were determined according to Bradford [63] using Quick Start™ Bradford Protein Assay (Bio-Rad Laboratories GmbH; Puchheim, Germany) according to the manufacturer’s specifications. The absorbance at 595 nm was measured with a TECAN Spark (Männedorf, Switzerland).

1 unit of CAD activity is defined as 1 µmol of IA produced in 1 min per mg of protein in the crude cell extract.

### Processing of pyrolysis water

The PW was processed according to Kubisch and Ochsenreither [47] prior to cultivation. Potassium hydroxide was used to adjust the pH of 500 mL PW to 6.5–7. Afterwards, the PW was centrifuged at 4000 g for 30 min. The supernatant was filtered with a funnel filter. The filtered PW was vacuum concentrated with a rotary evaporator ‘Hei-VAP Core’ (Heidolph Instruments GmbH & Co. KG; Kelheim, Germany) at 80 °C. The pressure was reduced every 30 min from 400 to 200 mbar and maintained at that pressure for 4 h. The remaining solid was resuspended in 500 mL deionized water and sonicated for 30 min. The pH was further increased to 13 by adding solid potassium hydroxide and the PW was boiled in a closed bottle at 100 °C for 2 h. After cooling to RT, the processed PW was filtered with a funnel filter and neutralized by addition of 96% (w/v) H_2_SO_4_. After repeated filtration, 10% (w/v) activated carbon was mixed with PW on a magnetic stirrer and incubated for 10 min at RT. The activated carbon was separated from the PW by centrifugation at 4000 g for 30 min at RT. The supernatant was filtered as described above and sterile filtered with a 0.2 µm PES membrane filtration cup (VWR; Darmstadt, Germany). The processed PW was stored at 4 °C until further use.

### Statistics

All data presented in this study show mean and standard deviation of at least three biological replicates. A biological replicate is defined as the independent cultivation of a single colony picked from a freshly streaked plate in separately prepared media. Whenever possible, three positive transformants of independent transformation events were isolated and used for respective cultivations. Significance was determined in Microsoft Office Excel Professional Plus 2016 using a two-sample *t*-test assuming the same variance.

## Supplementary Information

**Additional file 1: Figure S1.** Growth of *C. glutamicum* wild type in CGXII minimal medium with 20 g acetate L^-1^ without itaconate (black circles), 50 mM (green triangles) or 250 mM (green diamonds) potassium itaconate. Cultures supplemented with 100 mM (grey triangles) and 500 mM (grey diamonds) of KCl were included as controls to show the effect of the potassium ions on growth. All cultures were cultivated in shaking flasks at 30 °C.

**Additional file 2: Table S1.** Strains used in this study. **Table S2.** Plasmids used in this study. **Table S3**. Primers used in this study.

## Data Availability

All data generated or analyzed during this study are included in this published article and its supplementary information file.
