# Supplementary material for: Metabolic engineering of Corynebacterium glutamicum for acetate-based itaconic acid production
Source: Biotechnol Biofuels Bioprod. 2022 Dec 14;15:139. doi: 10.1186/s13068-022-02238-3 (PMC9753420; doi:10.1186/s13068-022-02238-3)
Supplement: Supplementary file 1 — Additional file 1: Figure S1. Growth of C. glutamicum wild type in CGXII minimal medium with 20 g acetate L-1 without itaconate (black circles), 50 mM (green triangles) or 250 mM (green diamonds) potassium itaconate. Cultures supplemented with 100 mM (grey triangles) and 500 mM (grey diamonds) of KCl were included as controls to show the effect of the potassium ions on growth. All cultures were cultivated in shaking flasks at 30 °C. [file 13068_2022_2238_MOESM1_ESM.docx]

**Figure S1**. Growth of *C. glutamicum* wild type in CGXII minimal medium with 20 g acetate L^-1^ without itaconate (black circles), 50 mM (green triangles) or 250 mM (green diamonds) potassium itaconate. Cultures supplemented with 100 mM (grey triangles) and 500 mM (grey diamonds) of KCl were included as controls to show the effect of the potassium ions on growth. All cultures were cultivated in shaking flasks at 30 °C.
