# Supplementary material for: Metabolic engineering of Corynebacterium glutamicum for acetate-based itaconic acid production
Source: Biotechnol Biofuels Bioprod. 2022 Dec 14;15:139. doi: 10.1186/s13068-022-02238-3 (PMC9753420; doi:10.1186/s13068-022-02238-3)
Supplement: Supplementary file 2 — Additional file 2: Table S1. Strains used in this study. Table S2. Plasmids used in this study. Table S3. Primers used in this study. [file 13068_2022_2238_MOESM2_ESM.docx]

**Table S1.** Strains used in this study.

| No. | Strains | Relevant characteristics | Reference |
| --- | --- | --- | --- |
| S1 | *C. glutamicum* ATCC 13032 | Wild type | (1, 2) |
| S2 | *E. coli* DH5α | F^–^ φ80*lac*ZΔM15 Δ(*lac*ZYA-*arg*F)U169 *rec*A1 *end*A1 *hsd*R17(r_K_^–^, m_K_^+^) *pho*A *sup*E44 λ^–^*thi*-1 *gyr*A96 *rel*A1 | (3) |
| S3 | *C. glutamicum* IDH^A94D^ | Expression of an isocitrate dehydrogenase (IDH) variant with 10% of wild type activity | This work |
| S4 | *C. glutamicum* IDH^G407S^ | Expression of an IDH variant with 55% of wild type activity | This work |
| S5 | *C. glutamicum* IDH^R453C^ | Expression of an IDH variant with 29% of wild type activity | This work |
| S6 | *C. glutamicum* IDH^R453C^  ΔP*_pck_*::P*_dapA_*-A8 | Expression of an IDH variant with 29% of wild type activity. Exchange of the native *pck* promoter with the *dapA*-A8 promoter. | This work |
| S7 | *C. glutamicum* IDH^R453C^  ΔP*_pck_*::P*_dapA_*-A16 | Expression of an IDH variant with 29% of wild type activity. Exchange of the native *pck* promoter with the *dapA*-A16 promoter. | This work |
| S8 | *C. glutamicum* IDH^R453C^  ΔP*_pck_*::P*_dapA_*-C5 | Expression of an IDH variant with 29% of wild type activity. Exchange of the native *pck* promoter with the *dapA*-C5 promoter. | This work |
| S9 | *C. glutamicum* IDH^R453C^  ΔP*_pck_::*P*_dapA_*-C7 | Expression of an IDH variant with 29% of wild type activity. Exchange of the native *pck* promoter with the *dapA*-C7 promoter. | This work |
| S10 | *C. glutamicum* Δ*ramB* | Deletion of the *ramB* gene encoding the transcriptional regulator RamB | (4) |
| S11 | *C. glutamicum* Δ*ramB* Δ*glnE* | *C. glutamicum* Δ*ramB* with additional deletion of the glnE gene encoding the adenylyltransferase GlnE. | This work |
| S12 | *C. glutamicum* Δ*ramB* Δ*gdh* | *C. glutamicum* Δ*ramB* with additional deletion of the *gdh* gene encoding glutamate dehydrogenase. | This work |
| S13 | *C. glutamicum* Δ*ramB* Δ*gdh* ICD^R453C^ | *C. glutamicum* Δ*ramB* Δ*gdh* expressing an IDH variant with 29% of wild type activity | This work |

**Table S2.** Plasmids used in this study.

| No. | Plasmids,  purpose | Restriction of plasmid | Features or insert | Reference |
| --- | --- | --- | --- | --- |
| P1 | pEKEx2  *C. glutamicum E. coli* shuttle vector | - | *Kan^R^*, P_tac_, *lacI*^q^, *oriV_.g.G_*, *oriV_E.c._* | (5) |
| P2 | pK19*mobsacB*  Vector for chromosomal mutation in *C. glutamicum* | - | *Kan^R^, oriV, sacB, lacZ* | (6) |
| P3 | pEKEx2_*malEcad*_opt_ | - | - | (7) |
| P4 | pEKEx2_*malEcad*_opt_-*ramA*  Construction of *C.g.* ITA10 | P3 cut with *EcoRI* | *ramA* amplified from S1 gDNA with O1 + O2 | This work |
| P5 | pK19*mobsacB*_IDH^A94D^  Construction of *C.g.* S3 | - | - | (8) |
| P6 | pK19*mobsacB*_IDH^G407S^  Construction of *C.g.* S4 | - | - | (8) |
| P7 | pK19*mobsacB*_IDH^R453C^  Construction of *C.g.* S5, S13 | - | - | (8) |
| P8 | pK19*mobsacB*_ΔP*_gltA_*::P*_dapA_*-A8  PCR template to construct P12 | - | - | (9) |
| P9 | pK19*mobsacB*_ΔP*_gltA_*::P*_dapA_*-A16  PCR template to construct P13 | - | - | (9) |
| P10 | pK19*mobsacB*_ΔP*_gltA_*::P*_dapA_*-C5  PCR template to construct P14 | - | - | (9) |
| P11 | pK19*mobsacB*_ΔP*_gltA_*::P*_dapA_*-C7  PCR template to construct P15 | - | - | (9) |
| P12 | pK19*mobsacB*_ΔP*_pck_*::P*_dapA_*-A8  Construction of *C.g.* S6 | P2 cut with *EcoRI* and *BamHI* | Flank 1 and Flank 2 amplified from S1 gDNA with O8 + O9 and O10 + O11, respectively. Promoter fragment amplified from P8 with O12+O13 | This work |
| P13 | pK19*mobsacB*_ΔP*_pck_*::P*_dapA_*-A16  Construction of *C.g.* S7 | P2 cut with *EcoRI* and *BamHI* | Flank 1 and Flank 2 amplified from S1 gDNA with O8 + O9 and O10 + O11, respectively. Promoter fragment amplified from P9 with O12+O13 | This work |
| P14 | pK19*mobsacB*_ΔP*_pck_*::P*_dapA_*-C5  Construction of *C.g.* S8 | P2 cut with *EcoRI* and *BamHI* | Flank 1 and Flank 2 amplified from S1 gDNA with O8 + O9 and O10 + O11, respectively. Promoter fragment amplified from P10 with O12+O13 | This work |
| P15 | pK19*mobsacB*_ΔP*_pck_*::P*_dapA_*-C7  Construction of *C.g.* S9 | P2 cut with *EcoRI* and *BamHI* | Flank 1 and Flank 2 amplified from S1 gDNA with O8 + O9 and O10 + O11, respectively. Promoter fragment amplified from P11 with O12+O13 | This work |
| P16 | pK19*mobsacB*_Δ*glnE*  Construction of *C.g.* S11 | P2 cut with *EcoRI* and *BamHI* | Flank 1 and Flank 2 amplified with O16 + O17 and O18 + O19 | This work |
| P17 | pK19*mobsacB*_Δ*gdh*  Construction of *C.g.* S12 | P2 cut with *EcoRI* and *BamHI* | Flank 1 and Flank 2 amplified with O20 + O21 and O22 + O23 | This work |

**Table S3.** Primers used in this study. Binding region in bold.

| No. | Sequence 5‘-3‘ | | | Purpose |
| --- | --- | --- | --- | --- |
| O1 | TGAAGTCCCCACTGGTGTAAGAATTTCCTGCAGAAGGAGATCA**GTGGATACCCAGCGGATTAAAG** | | | Amplification of *ramA* |
| O2 | GCTGTAAAACGACGGCCAGTGAATTC**TTAAGGCAGTGCGCCGATCC** | | | Amplification of *ramA* |
| O3 | **GCTACGGCGTTTCACTTCTG** | | | Sequencing of P4 |
| O4 | **GAACGCATCCTGCACAAGTACC** | | | sequencing of P4 |
| O5 | **TTACTTCTTCAGTGCGTCAACG** | | | Sequencing of P5,6,7 |
| O6 | **ATGGCTAAGATCATCTGGACC** | | | Sequencing of P5,6,7 |
| O7 | **TGTAGTCACGCAGAACGTTACC** | | | Sequencing of P5,6,7 |
| O8 | CAGGTCGACTCTAGAGGATCC**TTGCCCTCTGGGTTGATC** | | | Amplification of *pck-*flank 1 |
| O9 | TAAGACCGGAGCCGCTCGAG**AAATCTGGAGAAGTAATGACTACTG** | | | Amplification of pck flank 1 |
| O10 | TAACTGCAGAACCAATGCAT**CTGCCATCACAATCCAAGC** | | | Amplification of *pck-*flank 2 |
| O11 | GAGCTCGGTACCCGGGGATCC**CACAAACATAAATCCCCACAG** | | | Amplification of *pck-*flank 2 |
| O12 | TGCTTGGATTGTGATGGCAG**ATGCATTGGTTCTGCAGTTATC** | | | Amplification of P*_dapA_* |
| O13 | GTCATTACTTCTCCAGATTT**CTCGAGCGGCTCCG** | | | Amplification of P*_dapA_* |
| O14 | **CGTTACCCCAAAGGTTATATCC** | | | Sequencing of P12, P13, P14, P15 |
| O15 | **GGATATAACCTTTGGGGTAACG** | | | Sequencing of P12, P13, P14, P15 |
| O16 | TTGGGGCTCCCACCACCTAC**AGCTTCTCCCCTCCTTC** | | | Amplification of flank 1 *glnE* + sequencing |
| O17 | CCTGCAGGTCGACTCTAGAGGATC**ATGCGCTCACCATGGCCGACAAC** | | | Amplification of  flank 1 *glnE* + sequencing |
| O18 | GATGAAGGAGGGGAGAAGCT**GTAGGTGGTGGGAGCC** | | | Amplification of  flank 2 *glnE* + sequencing |
| O19 | | GTTGTAAAACGACGGCCAGTGAATT**ATCAGATGCTTCATCCAGC** | Amplification of  flank 2 *glnE* + sequencing | |
| O20 | | GTCGACTCTAGAGGATCCCC**CAACTACGGTCTGGAACCATCAC** | Amplification of  flank 1 *gdh* | |
| O21 | | GAGATGGGAACGAGGAAATC**GACCCCTGCGCTTTACTTAAACC** | Amplification of  flank 1 *gdh* | |
| O22 | | **GATTTCCTCGTTCCCATCTCGG** | Amplification of flank 2 *gdh* | |
| O23 | | TGAATTCGAGCTCGGTACCC**TCGTACCAATTCCATTTGAGGGC** | Amplification of  flank 2 *gdh* | |
| O24 | | **TTCCAGTCAGCGCAAAGGG** | Sequencing of P17 | |
| O25 | | **CGGTCGCCCAATTGAGGAG** | Sequencing of P17 | |

References

1. Abe S, Takayama K-I, Kinoshita S. Taxonomical studies on glutamic acid-producing bacteria. The Journal of General and Applied Microbiology. 1967;13(3):279-301.

2. Ikeda M, Nakagawa S. The *Corynebacterium glutamicum* genome: features and impacts on biotechnological processes. Applied Microbiology and Biotechnology. 2003;62(2):99-109.

3. Hanahan D. Studies on transformation of *Escherichia coli* with plasmids. Journal of Molecular Biology. 1983;166(4):557-80.

4. Gerstmeir R, Cramer A, Dangel P, Schaffer S, Eikmanns BJ. RamB, a novel transcriptional regulator of genes involved in acetate metabolism of *Corynebacterium glutamicum*. Journal of Bacteriology. 2004;186(9):2798-809.

5. Eikmanns BJ, Thum-Schmitz N, Eggeling L, Lüdtke K-U, Sahm H. Nucleotide sequence, expression and transcriptional analysis of the *Corynebacterium glutamicum* gltA gene encoding citrate synthase. Microbiology. 1994;140(8):1817-28.

6. Schäfer A, Tauch A, Jäger W, Kalinowski J, Thierbach G, Pühler A. Small mobilizable multi-purpose cloning vectors derived from the *Escherichia coli plasmids* pK18 and pK19: selection of defined deletions in the chromosome of *Corynebacterium glutamicum*. Gene. 1994;145(1):69-73.

7. Otten A, Brocker M, Bott M. Metabolic engineering of *Corynebacterium glutamicum* for the production of itaconate. Metabolic Engineering. 2015;30:156-65.

8. Schwentner A, Feith A, Münch E, Busche T, Rückert C, Kalinowski J, et al. Metabolic engineering to guide evolution–Creating a novel mode for L-valine production with *Corynebacterium glutamicum*. Metabolic Engineering. 2018;47:31-41.

9. van Ooyen J, Noack S, Bott M, Reth A, Eggeling L. Improved L‐lysine production with *Corynebacterium glutamicum* and systemic insight into citrate synthase flux and activity. Biotechnology and Bioengineering. 2012;109(8):2070-81.
